# Supplementary material for: The feasibility of soluble Fms-Like Tyrosine kinase-1 (sFLT-1) and Placental Growth Factor (PlGF) ratio biomarker in predicting preeclampsia and adverse pregnancy outcomes among medium to high risk mothers in Kuala Lumpur, Malaysia
Source: PLoS One. 2022 Mar 11;17(3):e0265080. doi: 10.1371/journal.pone.0265080 (PMC8916650; doi:10.1371/journal.pone.0265080)
Supplement: Attachment — Submitted filename: Response to Reviewers_29jan22.pdf [file pone.0265080.s005.pdf]

UNIVERSITI KEBANGSAAN MALAYSIA
The National University of Malaysia

Jabatan Kesihatan Masyarakat *Department of Community Health*

29th January 2022

Dear

Dr. Muhammad Tarek Abdel Ghafar, M.D
Academic Editor
PLOS ONE

Thank you for inviting us to submit a revised draft of our manuscript entitled, *"The feasibility of Soluble Fms-Like Tyrosine Kinase-1 (sFLT-1) and Placental Growth Factor (PlGF) ratio biomarker in predicting preeclampsia and adverse pregnancy outcomes among medium to high-risk mothers in Kuala Lumpur, Malaysia",* manuscript ID: PONE-D-21-25053R3 to PLOS ONE. We also appreciate the time and effort you and each of the reviewers have dedicated to providing insightful feedback on ways to strengthen our paper. Thus, it is with great pleasure that we resubmit our article for further consideration. We have incorporated changes that reflect the detailed suggestions you have graciously provided.

To facilitate your review of our revisions, the following is a point-by-point response to the questions and comments delivered in your E-mail dated 20 January 2022.

We agreed to add a minimal dataset and upload it as a Supporting Information file.

We hope that the editorial board will agree on the interest of this study.

Thank you.

Sincerely,

AP DR ROSNAH SUTAN
Department of Community Health, UKM Medical Centre

On behalf of all authors.

EDITOR SUGGESTIONS:

Journal Requirements:

1. *Table 2: Gravida classification is not significant in univariate analysis, so it should not be included in multivariate analysis.*

    - **<u>RESPONSE:</u> We have removed gravida classification in multivariate analysis and reanalyzed. The new result from multivariate analysis was updated in Table 2 (page 12,line 222, 236-238), Abstract(page 3, line 40 to 45, 48-49, 51-52) and in discussion part(page 20, line 335-337)**

2. Table 3: Adjusted OR of Low birth weight, premature delivery, and NICU admission should be added.

    - **<u>RESPONSE:</u> We have added Adjusted OR of Low birth weight, premature delivery, and NICU admission in Table 3 (page 15-16, line 253) and Table 4(page 17, line 276) and description at page 14 line 250**

3. Abbreviations such as PPV and NPV should be defined in full-term before their first mention in the abstract.

    - **RESPONSE: We have defined full-term of PPV and NPV abbreviations before their first mention in the abstract (page 3).**

UKM
