# Supplementary material for: The feasibility of soluble Fms-Like Tyrosine kinase-1 (sFLT-1) and Placental Growth Factor (PlGF) ratio biomarker in predicting preeclampsia and adverse pregnancy outcomes among medium to high risk mothers in Kuala Lumpur, Malaysia
Source: PLoS One. 2022 Mar 11;17(3):e0265080. doi: 10.1371/journal.pone.0265080 (PMC8916650; doi:10.1371/journal.pone.0265080)
Supplement: Attachment — Submitted filename: Response to Reviewers.doc [file pone.0265080.s002.doc]

29^th^ September 2021

Dear

Dr. Muhammad Tarek Abdel Ghafar, M.D

Academic Editor

PLOS ONE

Thank you for inviting us to submit a revised draft of our manuscript entitled, *"The feasibility of Soluble Fms-Like Tyrosine Kinase-1 (sFLT-1) and Placental Growth Factor (PlGF) ratio biomarker in predicting preeclampsia and adverse pregnancy outcomes among medium to high risk mothers",* manuscript ID: PONE-D-21-25053 to PLOS ONE. We also appreciate the time and effort you and each of the reviewers have dedicated to providing insightful feedback on ways to strengthen our paper. Thus, it is with great pleasure that we resubmit our article for further consideration. We have incorporated changes that reflect the detailed suggestions you have graciously provided.

To facilitate your review of our revisions, the following is a point-by-point response to the questions and comments delivered in your E-mail dated 14 September 2021.

We hope that the editorial board will agree on the interest of this study.

Thank you.

Sincerely,

**AP DR ROSNAH SUTAN**

Department of Community Health, UKM Medical Centre

On behalf of all authors.

**EDITOR SUGGESTIONS:**

**Journal Requirements:**

1. *Please ensure that your manuscript meets PLOS ONE's style requirements, including those for file naming.*
   - **RESPONSE: File naming was edited to comply with the style requirements. We hope it is now correct and complies with the style requirements.**
2. *During your revisions, please note that a simple title correction is required: please add the location at which the study has been undertaken (i.e.* ***The feasibility of Soluble Fms-Like Tyrosine Kinase-1 (sFLT-1) and Placental Growth Factor (PlGF) ratio biomarker in predicting preeclampsia and adverse pregnancy outcomes among medium to high risk mothers in Kuala Lumpur, Malaysia****). Please ensure this is updated in the manuscript file and the online submission information.*

- **RESPONSE: Thank you for providing these insights. We have corrected the manuscript title as per suggestion and updated in the manuscript file and the online submission information.**

3. *Thank you for stating the following in the Competing Interests section:*

***Funding***

*This research received funding through the Fundamental Grant (FF-2019-371) and Matching Grant (FF-2019-371/1) from Universiti Kebangsaan Malaysia.*

***Conflict of Interest***

*Provision of Elecsys sFlt-1/PlGF and PreciControl Multimaker test kits from Roche Diagnostics International Ltd is acknowledged.*

*The funders had no role in study design, data collection and analysis, decision to publish, or preparation of the manuscript.*

*Please confirm that this does not alter your adherence to all PLOS ONE policies on sharing data and materials, by including the following statement: "This does not alter our adherence to PLOS ONE policies on sharing data and materials.” (as detailed online in our guide for authors http://journals.plos.org/plosone/s/competing-interests). If there are restrictions on sharing of data and/or materials, please state these. Please note that we cannot proceed with consideration of your article until this information has been declared.*

*Please include your updated Competing Interests statement in your cover letter; we will change the online submission form on your behalf.*

- **RESPONSE: We have included the following statement *"This does not alter our adherence to PLOS ONE policies on sharing data and materials” as* an updated Competing Interests statement in the cover letter**

4. *Please ensure that you include a title page within your main document. You should list all authors and all affiliations as per our author instructions and clearly indicate the corresponding author.*

- **RESPONSE: We have included the title page within the main document. We have listed all authors and all affiliations as per the author instructions and indicated the corresponding author.**

5. *Please include a separate caption for each figure in your manuscript.*

*•*	**RESPONSE: We have included a separate caption for each figure in the main manuscript body**

**REVIEWER #1 COMMENTS:**

*Generally, the design of this study is well attempted and these results must be helpful to the pregnant women in Malaysia. Interestingly, the occupation may impact on the development of preeclampsia in Malaysian.*

6. *In the algorithm of this study, some population may change the results of sFLT-1/PlGF ratio, positive to negative, but clinically the development of preeclampsia seems ‘one way’. If some rare cases exist in this study, please tell the number and the process or outcome of them*.
   - **RESPONSE: That is an interesting query.** **However, there were no positive case change to negative result in our sample population.**
7. *In Table 2, according to the sentence, line 157, you should correct the small title of ‘History of PIH’ to ‘History of HDP’.*
   - **RESPONSE: Thank you for this observation. I have corrected the small title of ‘History of PIH’ to ‘History of HDP’ in Table 2 in the revised manuscript.**
8. *In Fig. 3 you showed the wrong number in the major title. Not Fig. 2 but Fig. 3.*
   - **RESPONSE:** **Our apologies for the mistake. We have included a new Figure 3 with the correct title.**
9. *In Table 4, you also have to correct the title, “1 minute’ to ‘5 minutes”*
   - **RESPONSE: Thank you for this observation. I have corrected the title, “1 minute’ to ‘5 minutes” in Table 4.**

**REVIEWER #2 COMMENTS:**

*In this paper, the authors evaluated the feasibility of the sFLT-1/PlGF biomarker ratio in predicting preeclampsia and adverse pregnancy outcomes using a single cut-off point of >38 among mothers in Malaysia. The study confirms that although sFLT-1/PlGF ratio is a feasible biomarker for predicting pregnancy, a combined approach with maternal clinical characteristics provides a better prediction for preeclampsia and adverse pregnancy outcomes.*

10. Specific Areas:

*Introduction
Language editing is recommended at line 41 of the manuscript.*

- **RESPONSE: We agree with your assessment. Language editing has been performed as in lines 57-59 in the revised manuscript, as follows: “**Identifying women at risk of developing preeclampsia for early intervention with commencement of aspirin/calcium as prophylaxis, close monitoring, and timely delivery are critical steps in the management to prevent adverse pregnancy outcomes.”**.**

*Materials and Methods:
Authors should indicate the full meaning of the abbreviation, ISSHP, at the first mention in the write up at line 113 of the manuscript.*

- **RESPONSE: Thank you for this suggestion. We have stated in full the meaning of the abbreviation ISSHP on page 7, lines 134-135, as “International Society for the Study of Hypertension in Pregnancy”.**

*Results and Discussion
Authors indicated that 57% of the study participants completed the research algorithm. Were there reasons or factors that explain why a significant number were lost to follow up especially after the first test?*

- **RESPONSE: You have raised an important point.** There are several reasons to explain why a significant number of study participants were lost to follow up especially after the first test, including the government enforcement of the **movement control order in response to the COVID-19 pandemic, work and family commitments, and logistic issues. We have included this point in the Discussion Section in Lines 363-366.**

*Authors should kindly be consistent with referencing style and not mix Harvard with Vancouver as identified at line 293-294 and 310.*

- **RESPONSE: We has edited the referencing style to Vancouver format as in lines 320-321 and 339.**

***REVIEWER #3*** ***COMMENTS****:*

*The author analyzes the relationship between the sFlt-1 / PlGF ratio and preeclampsia in detail. The content is very interesting and orderly. But I'm not sure what's new.　That is the reason I don't think it is suitable for this journal.　This study is very interesting, so I would be happy if you rewrite the conclusions so that you can easily understand what is new.*

- **RESPONSE: Thank you for providing these useful insights. We** **has** **rewritten the conclusions and highlighted the new perspectives of this study as in lines 45-48, 278-280 and 369-373.**
