# Supplementary material for: The feasibility of soluble Fms-Like Tyrosine kinase-1 (sFLT-1) and Placental Growth Factor (PlGF) ratio biomarker in predicting preeclampsia and adverse pregnancy outcomes among medium to high risk mothers in Kuala Lumpur, Malaysia
Source: PLoS One. 2022 Mar 11;17(3):e0265080. doi: 10.1371/journal.pone.0265080 (PMC8916650; doi:10.1371/journal.pone.0265080)
Supplement: Attachment — Submitted filename: Response to Reviewers_15nov21.doc [file pone.0265080.s003.doc]

15^th^ November 2021

Dear

Dr. Muhammad Tarek Abdel Ghafar, M.D

Academic Editor

PLOS ONE

Thank you for inviting us to submit a revised draft of our manuscript entitled, *"The feasibility of Soluble Fms-Like Tyrosine Kinase-1 (sFLT-1) and Placental Growth Factor (PlGF) ratio biomarker in predicting preeclampsia and adverse pregnancy outcomes among medium to high-risk mothers in Kuala Lumpur, Malaysia",* manuscript ID: PONE-D-21-25053R1 to PLOS ONE. We also appreciate the time and effort you and each of the reviewers have dedicated to providing insightful feedback on ways to strengthen our paper. Thus, it is with great pleasure that we resubmit our article for further consideration. We have incorporated changes that reflect the detailed suggestions you have graciously provided.

To facilitate your review of our revisions, the following is a point-by-point response to the questions and comments delivered in your E-mail dated 19 October 2021.

We hope that the editorial board will agree on the interest of this study.

Thank you.

Sincerely,

AP DR ROSNAH SUTAN

Department of Community Health, UKM Medical Centre

On behalf of all authors.

**EDITOR SUGGESTIONS:**

**Journal Requirements:**

1. *Please review your reference list to ensure that it is complete and correct. If you have cited papers that have been retracted, please include the rationale for doing so in the manuscript text or remove these references and replace them with relevant current references. Any changes to the reference list should be mentioned in the rebuttal letter that accompanies your revised manuscript. If you need to cite a retracted article, indicate the article’s retracted status in the References list and also include a citation and full reference for the retraction notice.*
   - **RESPONSE: We have reviewed the references. Therefore, we made some changes as stated below.**
     (i) **There are few duplicate references:**
         (1) **References number 60 and 61. We have deleted reference number 61 and replaced it with citation number 60.**
         (2) **References number 58 and 59. We have deleted reference number 59 and replaced it with citation number 58.**
         (3) **References number 32 and 45. We have deleted reference number 45 and replaced it with citation number 32.**
         (4) **We renumbered again to make it align in both manuscript body and in the reference list.**
     (ii) **We don’t cite any papers that have been retracted.**

**REVIEWER #2 COMMENTS:**

*Reviewer #2: Initial comments have all been addressed by authors.*

*Below are few additional comments for authors to address.*

*1. Authors should kindly review the definition of preeclampsia to be “Hypertension with proteinuria and/or maternal organ damage…” rather than 'or' in the introduction and the entire write-up.*

- **RESPONSE: We agree with your assessment. We have changed the definition as below:**

**line 52 page 3 *“Preeclampsia is characterized by the combined presentation of hypertension with proteinuria and/or maternal organ dysfunction, such as renal insufficiency, liver involvement, neurological or haematological complications, and uteroplacental dysfunction as evidenced by fetal growth restriction (FGR) (2, 6)****.”*

**Line 131 page 7 *–*** *‘****hypertension with proteinuria and/or maternal organ dysfunction’***

2. *Per the Aspre trial by FMF group, optimal benefits of low dose aspirin are between 12-16 gestational weeks. It will be important to know from the authors if women who were high risk for preeclampsia (chronic hypertension, previous HDP, etc) were on aspirin before being recruited into the study. If not, were they started after 20 gestational weeks? This will be essential to know because it will be unethical or controversial to delay onset of aspirin in such high risk group. Is the practice of administering LD aspirin to such high risk women incorporated in the Malaysian Clinical Practice Guidelines (CPG)?*

**RESPONSE: You have raised an important point.** **Women who were high risk for preeclampsia were on aspirin before being recruited into this study. Our enrolment of respondents into the study starts at ≥20 weeks’ gestation until delivery (Line 105, page 6). The Malaysian Clinical Practice Guidelines (CPG) did incorporated practice of administering low dose aspirin to high-risk women for preeclampsia such as:**

- **Hypertensive disease during previous pregnancy**
- **Chronic kidney disease**
- **Autoimmune disease such as Systemic Lupus Erythematosus (SLE) or anti-phospholipid syndrome (APS)**
- **Type 1 or type 2 diabetes mellitus**
- **Chronic hypertension**

**We did not delay or alter any management as written in the CPG.**
